# Using Data Crawlers and Semantic Web to Build Financial XBRL Data Generators: The SONAR Extension Approach

**DOI:** 10.1155/2014/506740

**Published:** 2014-01-23

**Authors:** Miguel Ángel Rodríguez-García, Alejandro Rodríguez-González, Ricardo Colomo-Palacios, Rafael Valencia-García, Juan Miguel Gómez-Berbís, Francisco García-Sánchez

**Affiliations:** ^1^Department of Informatics and Systems, University of Murcia, Espinardo, 30100 Murcia, Spain; ^2^Bioinformatics at Centre for Plant Biotechnology and Genomics UPM-INIA, Polytechnic University of Madrid, Pozuelo de Alarcón, 28223 Madrid, Spain; ^3^Computer Science Department, Carlos III University of Madrid, Leganés, 28911 Madrid, Spain

## Abstract

Precise, reliable and real-time financial information is critical for added-value financial services after the economic turmoil from which markets are still struggling to recover. Since the Web has become the most significant data source, intelligent crawlers based on Semantic Technologies have become trailblazers in the search of knowledge combining natural language processing and ontology engineering techniques. In this paper, we present the SONAR extension approach, which will leverage the potential of knowledge representation by extracting, managing, and turning scarce and disperse financial information into well-classified, structured, and widely used XBRL format-oriented knowledge, strongly supported by a proof-of-concept implementation and a thorough evaluation of the benefits of the approach.

## 1. Introduction

The ability to acquire, communicate, and disseminate business information is vital for investor and management decision making [1]. Investors increasingly access basic financial information, such as annual and interim reports, and obtain timely information such as press releases, analysts' webcasts, and daily stock quotes, from corporate websites and other public information sources [2]. In this scenario, the online reporting of corporate events and actions in the websites of stock exchanges and other information intermediaries is becoming crucial for traders and managers around the globe [3]. Thus, according to Debreceny and Rahman [4], in comparison with quarterly reporting, this form of disclosure is considered to be more accurate for reporting price-sensitive information. Taking into account that the Internet underlying technologies have the power to revolutionize external reporting [5], a new practice has been born. This practice, known as Internet Financial Reporting can be defined as the distribution of corporate financial and performance information using Internet technologies such as the World Wide Web [6].

In the case of corporate sites, many relevant and recent works highlight their importance for corporate governance (e.g., [7, 8]). According to [9], a corporate website is essential for companies wishing to establish and maintain an international profile or access international sources of capital.

Kingstone et al. [10] state that the majority of International Financial Reporting (IFR) practices are voluntary and, mostly, unregulated. Many companies choose to voluntarily disseminate information on their corporate websites, although the extent of IFR varies significantly across firms ([6, 10, 11]). Due to the lack of standard format for communicating accounting information, organizations had to assemble it manually from often-incompatible information systems to prepare financial reports [12].

One recent IFR development is XBRL (Extensible Business Reporting Language), which is an XML based specification for efficient automated retrieval of financial information [13]. For example, corporate disclosures that are marked up with semantic XBRL tags allow users to quickly and seamlessly extract and compare information across companies [14]. Currently, XBRL is being promoted by the consortium XBRL International, which groups around 450 companies and organizations committed to extending the use of a standard taxonomy globally. XBRL reduces the costs associated with obtaining and analyzing information from businesses by addressing and eliminating incompatible reporting formats [15]. Moreover, using XBRL helps nonprofessional financial statement users acquire and integrate related financial statement and footnote information when making investment decisions [16]. The adoption and use of XBRL is expected to help avoid the extra effort and complications associated with multiple reconciliations between reporting formats [17].

However, XBRL adoption is still a challenge [1] and companies around the globe provide information using textual data that investors must analyze using manual methods. This fact is backed up by the works of Premuroso and Bhattacharya [18]. According to them, the current stage of development of XBRL also offers researchers significant opportunities as XBRL International grows in size, relevance and more firms globally start to report their financial results in the XBRL format.

This paper, following the path described in [17, 18], targets the building and testing of SONAR in a new environment. This initiative consists of a platform designed for information gathering using public sources and its transformation into XBRL format by means of the use of natural language processing and semantics.

The remainder of this paper is organized as follows.  Section 2 contains the literature review.  Section 3 discusses the main features of the approach, SONAR, the solution designed to extract information from public sources and convert it into XBRL format.  Section 4 presents the evaluation of SONAR, and Section 5 presents the conclusion, limitations, and areas for future research.

## 2. Background

In this section, authors briefly review two different research fields that integrate the SONAR approach: on the one hand, ontologies for knowledge representation in the Semantic Web and, on the other hand, human-computer interaction in the Semantic Web.

### 2.1. Ontologies for Knowledge Representation in the Semantic Web

The information contained in Web pages was originally designed to be human-readable. As the Web grows in both size and complexity, there is an increasing need for automating some of the time consuming tasks related to Web content processing and management. In 2001, Berners-Lee and his colleagues defined the Semantic Web as an extension of the current Web, in which information is given well-defined meaning, better enabling computers and people to work in cooperation [19]. The Semantic Web vision is based on the idea of explicitly providing the knowledge behind each Web resource in a manner that is machine processable. Ontologies [20] constitute the standard knowledge representation mechanism for the Semantic Web. During the last few years, a number of approaches have appeared with the purpose of structuring nonstructured and semistructured data sources. In particular, some approaches try to automatically associate data and semantic notes with HTML documents [21]. Other approaches focus on giving structure to semi-structured documents [22]. There are also approaches that attempt to automatically create an ontology from unstructured HTML documents [23].

Ontologies can be used to structure information. The formal semantics underlying ontology languages enables the automatic processing of the information in ontologies and allows the use of semantic reasoners to infer new knowledge. In this work, an ontology is seen as “a formal and explicit specification of a shared conceptualisation” [20]. Ontologies provide a formal, structured knowledge representation, with the advantage of being reusable and shareable. They also provide a common vocabulary for a domain and define, with different levels of formality, the meaning of the terms and the relations between them. Knowledge in ontologies is mainly formalized using five kinds of components: classes, relations, functions, axioms, and instances [24]. Classes in the ontology are usually organized into taxonomies. Sometimes the definition of ontologies has been diluted, in the sense that taxonomies are considered to be full ontologies [20]. In this work, the Ontology Web Language (OWL), which is the de facto Semantic Web standard language, has been used to represent the knowledge extracted from texts.

Creating and populating ontologies manually is a very time-consuming and labor-intensive task. Several methodologies have been designed in order to assist in building ontologies [25–27]. However, in order to overcome the bottleneck created by manually constructing ontologies [28], several (semi)automatic approaches are being researched. In this regard, it is necessary to differentiate between Ontology Learning [29] and Ontology Population [30]. Ontology Learning is about acquiring new knowledge in the form of concepts and relations to be added to an ontological model. As a consequence of this process, the inner structure of the ontology is modified. The goal of Ontology Population, on the other hand, is to extract and classify instances of the concepts and relations defined in an ontology from a particular data source. The process of Ontology Population does not change the structure of an ontology; what changes is the instances of concepts and relations in the domain. Instantiating ontologies with new knowledge is a relevant step towards the provision of valuable ontology-based knowledge services.

We can distinguish two types of ontology population: (i) ontology population from free text and (ii) ontology population from semistructured documents such as XML and HTML. In this work, a semiautomatic method for ontology population from semi-structured texts has been developed. Most of the information available on the Web is provided in terms of semistructured or unstructured HTML documents. Wrapping information from HTML tables has received much attention in the last few years [31]. This information is usually represented by means of databases [32] or is transformed into semantic annotations [10]. There are different approaches for populating ontologies from semi-structured or unstructured HTML documents. For example, in the work presented in [22] an ontology is populated using RDF triples obtained from HTML tables. Here, HTML documents are obtained from a Web Crawler and HTML tables are processed using wrappers based on predefined patterns. The Levenshtein distance [33] is used to identify which properties of the table are equivalent to the properties of concepts in the ontology, so they do not use any semantic information.

### 2.2. Human-Computer Interaction in the Semantic Web

In recent years, the utilization of natural language interfaces (NLIs) and controlled natural languages (CNLs) towards an effective human-computer interaction has received much attention in the context of the Semantic Web. Several platforms have been developed to function as either natural language ontology editors or natural language query systems. Two good examples in the first category are CNL Editor [34] (formerly OntoPath [35]) and GINO [36]. OntoPath is in fact situated in the frontier between these two categories because it manages and creates RDF ontologies, and it is also capable of defining queries from natural language sentences. It is composed of three main components in a layered architecture: “OntoPath-Syntax” in the syntax layer, “OntoPath-Object” in the object layer, and “OntoPath-Semantic” in the semantic layer. In the upper layer, a knowledge engineer and a domain expert can work together to define the domain ontology by using “OntoPath-Semantic.” Using this tool, it is possible to build a new ontology or edit a previously existing one. After defining a set of concepts and their corresponding relationships, the system returns the ontology in an RDF file. In the next layer, “OntoPath-Object” assists domain experts, who have no knowledge of ontologies, in graphically expressing natural language descriptions by using nodes and arcs that correspond to the elements in the ontology. This graphical description is then stored as RDF triples. Finally, in the lower layer, “OntoPath-Syntax” guides users in the query generation process through a simple, visual interface. The query is formed from the knowledge available in an ontology and is translated into RDF.

The ontology-based CNL editor extends OntoPath to provide a context-free grammar with lexical dependency for defining grammars. Using defined grammars, the CNL editor enables the system to get structured data from the writer narratives with sophisticated, pattern-aware, and informal expressions. Stemming from there, the editor provides guidance on the proper choice of words and translates the results into RDF triples. The architecture of the CNL editor consists of five components, namely, an interface, through which the system recommends proper next words to the writer; a parser, which processes an incoming sentence and determines the dependencies; a predictor, which examines the relations in the domain ontology to make a recommendation; a lexicon pool, which sends the candidate's next words to the interface; and a triple generator, which generates RDF triples when the sentence is completed.

GINO (Guided Input Natural Language Ontology Editor) allows users to edit and query any OWL knowledge base using a guided input natural language akin to English. The user inputs a query or sentence into a free form text field and, based on the grammar, the system incremental parser offers the possible completions of the user entry by presenting the user with choice pop-up boxes. These pop-up menus offer suggestions on how to complete a current word or what the next word might be. The GINO architecture consists of four parts: a grammar compiler, which generates the necessary dynamic grammar rules to extend the static part of the grammar; a partially dynamically generated multilevel grammar, which is used to specify the complete set of parser-wise questions/sentences and to construct the SPARQL statements from entered sentences; and an incremental parser, which maintains an in-memory structure representing all possible parse paths of the currently entered sequence of characters. Finally, the system also counts on an ontology access layer, implemented with Jena [37].

PANTO [38] and NLP-Reduce [39] are two representative examples in the category of natural language query systems. PANTO (Portable Natural Language Interface to Ontologies) is a system that takes ontologies and natural language queries as input and whose output is a series of SPARQL queries. When an ontology is selected as the underlying knowledge base, PANTO uses the so-called “Lexicon Builder” to automatically extract entities out of the ontology in order to build a lexicon. This lexicon is used to make sense of the words that appear in a natural language query. Once the user has entered a natural language query, PANTO produces a parse tree which is then translated into SPARQL. NLP-Reduce, on the other hand, is a domain-independent natural language interface for querying Semantic Web knowledge bases. Its architecture consists of five parts, namely: an interface, which allows the user to enter full natural language queries, sentence fragments or just keywords; a lexicon, which is automatically built by extracting all explicit and inferred subject-property object triples that exist in the knowledge base; an input query processor, which reduces a query by removing stop words and punctuation marks; a SPARQL query generator, which generates SPARQL queries from the input text; and an ontology access layer, which uses Jena and the Pellet reasoner [40].

In [41], other similar approaches are examined and the usefulness of NLIs is analyzed. The authors came to the conclusion that “casual end-users” strongly prefer querying using full-sentences rather than keywords or any other means. In [42], several related systems are analyzed and the exploitation of NLIs in a range of capabilities (e.g., the authoring of knowledge content, the retrieval of information from semantic repositories, and the generation of natural language texts from formal ontologies) is reviewed. In this report, the idea that CNLs could replace conventional Semantic Web ontologies was also explored but finally dismissed.

### 2.3. Financial Systems and XBRL Conversion Approaches

In a precise way, a financial system, in finance, is the system that allows the transfer of money between savers and borrowers [43]. However, from a computer science standpoint, we can consider that a financial system is any kind of information system which is applied to some branch of finance. With this consideration we can distinguish several kinds of financial systems with very different purposes like decision making [44], financial prediction [45], or financial search [46] among others.

The process of using the XBRL standard and, more concretely, of converting several formats to XBRL and vice versa is not a new task, but it can be seen that there are not too much efforts on this branch. One of the main aims when the researchers try to find ways to convert from custom formats to XBRL and vice versa is interoperability that the systems want to achieve between them [47, 48]. Several approaches have been designed like, for example, the effort made by Declerck and Krieger [49] with their design to translate in this case XBRL to DL (Description Logic) format. Other approaches pretend to make a transformation from XBRL to Linked Data [50].

## 3. The SONAR Approach: Core and Extensions

A large part of the huge volume of financial information that can be found in the World Wide Web is not annotated semantically. It can be found in a number of heterogeneous business sources and this information is characterized by unstructured content, disparate data models, and implicit knowledge. For this reason, it is important to build systems capable of gathering this information together and annotating it with enough accuracy to be used in other systems or applications, ideally using standards, such as XBRL.

**Figure 1:**
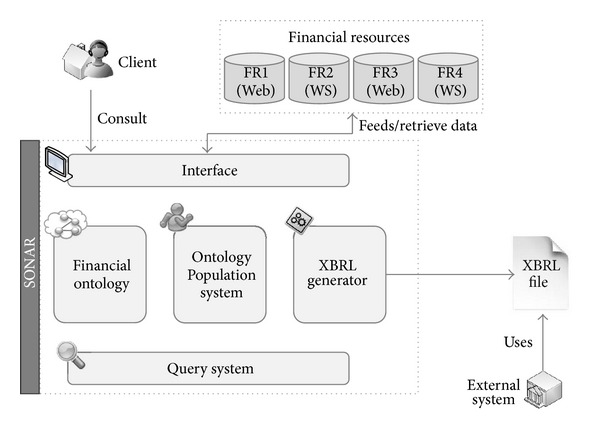
Architecture of the system.

Authors propose a set of technologies mixed in a single architecture to create a system capable of compiling this financial information, annotating them semantically following some financial patterns, and creating XBRL documents with the information obtained that can be used in automated environments to use the information stored in it. The main architecture of the system is shown in Figure 1.

In the next sections the main components of the architecture will be described as well as the relationships between them.

### 3.1. Ontology Population System

**Figure 2:**
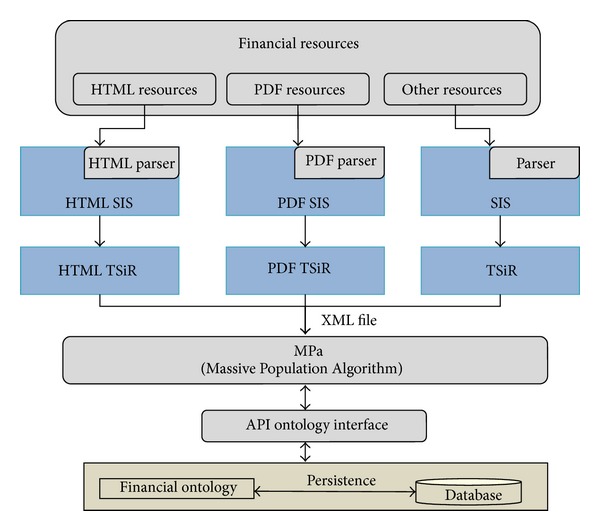
Architecture of the Ontology Population system.

The Ontology Population system [51] is capable of gathering knowledge from semi-structured and nonstructured texts. The ultimate goal of our approach is to populate an ontology with all the relevant information identified. The populated ontology will then serve as the keystone component for an up-to-date, knowledge-based search engine. The architecture of the proposed subsystem is shown in Figure 2. It is composed of three main components: (i) a set of selection systems (SIS), (ii) the “Selection and Converter System” (TSiR) module, and (iii) the “Massive Population Algorithm” (MPa) module. The input of the system is represented at the top of the figure. It consists of a collection of available Web information resources. The tool has been designed to support both semi-structured and non-structured texts. The module produces a number of ontology instances as outputs that are stored in the repository. The storage submodule is shown at the bottom of the figure.

In a nutshell, the system works as follows. Semi-structured or non-structured data sources available on the Internet are parsed to extract the information that can be gathered from the text. Currently, only semi-structured elements from HTML- and RSS-formatted documents are supported by the system. However, the platform can be easily extended to support other kinds of resources. In particular, both the tables contained in the HTML documents and the texts included in RSS documents constitute the semi-structured information used in this system. Users are shown the parts of the semi-structured texts identified by the parser. Then, users must choose which of the found elements are relevant and have to be stored in the knowledge base. Users have to set up two further parameters: (i) a set of substitution or transformation rules, which will be used by the TSiR module to transform the information into the appropriate format, and, optionally, (ii) the set of ontology concepts that are related to the information elements to be gathered from the source semi-structured text. This latter optional parameter aims to improve the efficiency and accuracy of the MPa module. Once users have indicated the tables from the resources in which they are interested, the TSIR module transforms the tables into an internal format in XML. For this purpose, the aforementioned user-defined transformation rules are applied. During this process, the position of the information in the tables is taken into account to form groups. Each group is represented in the form of tuples 〈attribute, literal〉. The XML file produced by the TSiR and the set of ontology concepts indicated by the user are the input of the MPa module. With this information, MPa generates the correspondences between the data in the semi-structured texts and the concepts in the ontology. Finally, the newly discovered ontology instances are stored in the knowledge base.

#### 3.1.1. Selection Information System (SIS)

The ultimate purpose of the proposed architecture is to make the ontology population algorithm independent from the data source, thus enabling the system to operate in a heterogeneous data space. The key to achieving this goal is to transform the information in these sources into a common representation format, which will be the input for the ontology population algorithm. The first essential step towards this end is to gather the information available in the documents that are being processed. This is precisely the aim of the “Selection Information System” (SIS) module.

**Figure 3:**
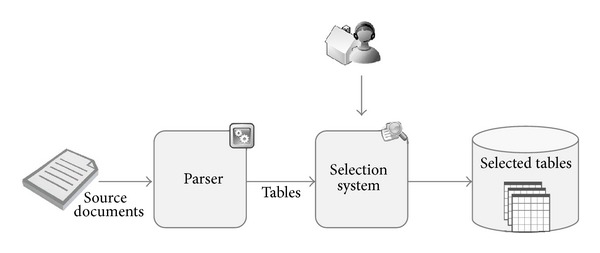
SIS Workflow.

At a preliminary stage, users must indicate the URLs of the sites that they want the system to analyze. An initial list of sites to process can also be established in the Web application configuration file. The way the SIS module works is shown in Figure 3. This component is responsible for assisting end-users in selecting the informational items to be analyzed. A SIS is necessary for each supported file format. At the current stage of development, the SIS subsystems make use of parsers, which focus on the discovery of the tables that are contained within the source documents. Up to now, parsers for HTML and PDF documents have been developed. Other semi-structured information sources such as RSS could also be easily incorporated into this scheme.

**Figure 4:**
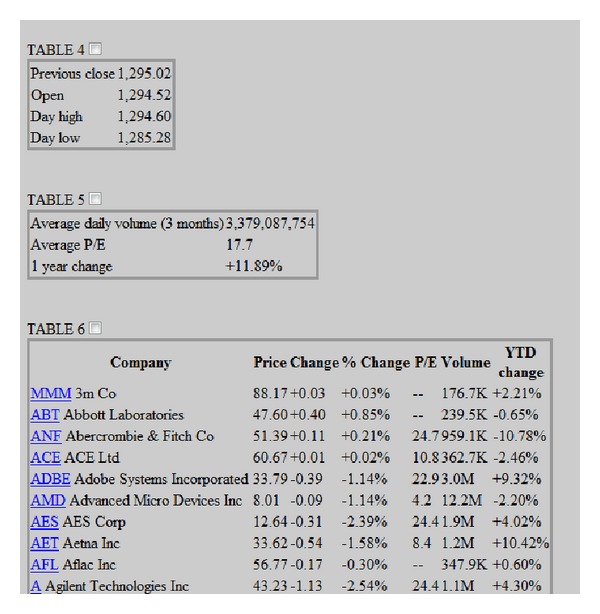
Screenshot of the list of retrieved tables.

In a second step, users are shown the list of tables identified by the parsers. Onwards, users must choose what tables to take into account for the next stages of the process. Consequently, end-users are the only stakeholders responsible for defining what has to be stored in the knowledge base. In Figure 4, a list of the tables retrieved by the system from the input Web page is depicted. By ticking the appropriate checkbox, the user is essentially asking the system to further process such table in order to extract the knowledge that is contained within it.

#### 3.1.2. Transform System Internal Representation (TSiR)

The TSiR is one of the key components of the architecture. It is responsible for transforming the tables, whatever their source is, into an internal representation format. This XML based representation will be common for all inputs and represents a unified format for the following stages of the process. A TSiR is necessary for each supported file format.

Next, the internal representation format is described and the way the system transforms the tables recognized by the SIS component into this common format is shown.

*(i) System Internal Representation*. This component makes use of a shared data structure for storing the information (in the form of tables) retrieved by the SIS modules. This data structure is an XML document whose syntax is given by an XML Schema (http://www.w3.org/XML/Schema). In a later stage, the Massive Population Algorithm (MPa) needs to receive a document complying with the referred XML Schema as input. In order to map a table into an XML file complying with the XML Schema, a first key step is the identification of the ontology classes (from the domain ontology) that are related to the table contents. This information, which is stored in the “classGroup” element of the XML file, is defined by the end-user and will be employed by the MPa module during the instance creation process. Once the ontology classes have been set, the system creates a “row” element in the XML file for each row within the table. After that, the attributes and their values are included.

The XML Schema defined to internally represent the information in tables is shown in Algorithm 1. One of the main advantages of making use of an XML Schema to represent the acceptable data structure is the possibility of handling the complying documents with the JAXB library (https://jaxb.java.net/). Fundamentally the goal is to be able to generate a set of Java classes based on the XML Schema and manage these classes instead of having to deal with the XML documents as such.

In this XML Schema, the two main elements are rows (“row” element) and groups of classes (“classGroup” element). Each “classGroup” element contains a set of ontology classes in the form of “classOntology” elements. A “classOntology” element refers to a class of the domain ontology. The “row” element represents a row in the input table. Each “row” is composed of a set of tuples (“tuple” element). Each “tuple” is defined by an attribute-value pair.

**Figure 5:**
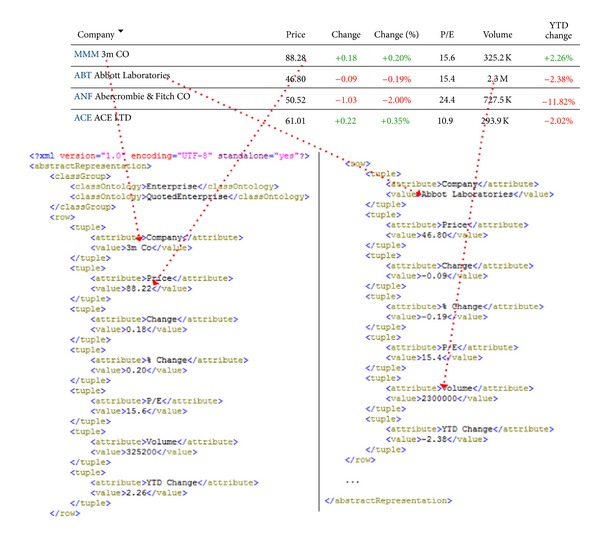
Mapping between a table and its corresponding XML file.

*(ii) From Tables to a Unified Representation Model*. The way a table is mapped into an XML file complying with the XML Schema described above is depicted in Figure 5. In this process, a first key step is the identification of the ontology classes (from the domain ontology) that are related to the table contents. This information, which is stored in the “classGroup” element of the XML file, is defined by the end-user and will be employed by the MPa module during the instance creation process. Once the ontology classes have been set, the system creates a “row” element in the XML file for each row within the table. The attributes and their values are included next.

**Figure 6:**
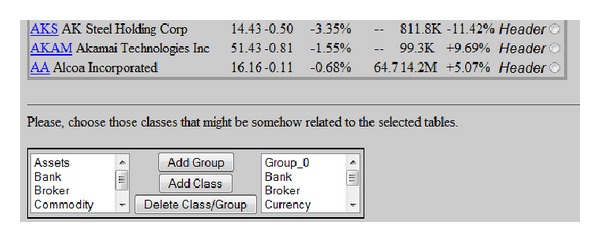
Screenshot of the selection of the relevant ontology classes.

Each row in an incoming table may result in a number of instances. Prior to creating an instance it is necessary to identify the ontology class to which the row under question refers. This association is generally carried out by the MPa subsystem. However, when the number of classes increases, the efficiency of the MPa drastically decreases. In order to overcome this shortcoming, users are asked to provide information about the ontology classes that may be involved in the tables by defining groups of classes as shown in Figure 6. Later, the MPa module will have to determine which group is associated with each row of the available tables.

#### 3.1.3. Massive Population Algorithm (MPa)

The MPa module is the main component of the Ontology Population system. It is in charge of creating ontology instances in accordance with the information in the tables and storing them in the knowledge base. The input of this component is the XML file generated by the TSiR. In a nutshell, the process that takes place within this component is as follows. First, a matching is produced to create the instances and decide the ontology classes to which they belong. Second, relations are established between the previously created instances. Finally, there is a consistency checking phase in which the system can identify contradictions. The ultimate goal of this component is to populate the ontology that underlies the knowledge-based decision support system. In the following, we provide a detailed description of how the instances are generated.

*(i) Matching.* In the matching phase, the instances related to the information obtained are created. For each XML row element, one or more instances can be created. A first step for this is to identify the group of classes that is the closest to the row under question. An affinity function is used to this end. The affinity of a row with a group of classes is the sum of the affinities of the row under consideration with each of the classes that belong to such group. Thus, let “*R*” be the row that is being processed and “*G*” the group of classes to which the row is being compared. The affinity between “*R*” and “*G*” is calculated as follows: if we have a group of classes “*G*” formed by “*n*” classes where *i* = (1,…, *n*), the affinity of a row “*R*” with *G* is

(1) $$\begin{array}{c} \text{AFFINITY}\left(R,G\right)=\sum_{i=1}^{n}\text{AFFINIT}{\text{Y}}^{*}\left(R,\text{Class}\left(i\right)\right), \end{array}$$

where by “Class *(i)*” (for *i* = 1 to *n*) represents each of the *n* ontology classes that belong to the group “*G*.” “AFFINITY*” is a function that takes into account the semantic annotations in the classes to measure the affinity between one given row and the classes. In Algorithm 2, the function performance and output are described.

The semantic annotations that the “AFFINITY*” function uses to calculate the closeness between a row and a class are defined in the ontology. Each concept (i.e., class of the ontology) and each attribute (i.e., datatype property of the ontology) have associated a semantic annotation consisting of a set of labels. These labels are used to define different names that may be given to the concept or attribute under question. The affinity between a row and a class is, thus, defined by the similarity between the attributes of each tuple that belong to the row with the labels of the class and its attributes.

*(ii) Ontology Population.* Each row of the input XML document can result in zero, one, or more instances. Once the system has recognized the group of classes that is more closely related to a particular row, the instances within the referred row must be created. However, while populating the ontology several issues must be taken into account. First, it is necessary to check that no other instance in the selected class contains the same information so that no data redundancy is present. Second, the relationships between the instances created during this process must be discovered. The processes related with (1) creating new instances, (2) avoiding data redundancy, and (3) establishing the relations between the instances are described next.

*(1) Instance Creation Algorithm*. The process of creating a new instance is shown in Algorithm 3. The function receives a row “*R*” and a group of classes “*G*” as input. Then, an instance is created for each ontology class within the group of classes. The datatype properties of the instances are set by comparing the labels in them with the attribute-value pairs that constitute each tuple in the row.

*(2) Redundancy*. Data redundancy can become a serious problem. Before a new instance is created, the existence of another instance that makes reference to the same concept should be checked. In OWL, the ontology language that is used in this work for knowledge representation, there is no primary key or anything similar that can uniquely identify each instance in the knowledge base. Thus, in order to determine whether two instances in the same class refer to the same concept, the values of both datatype and object properties of such instances must be considered.

However, comparing the value of each property for each instance in the knowledge base each time a new instance is to be created is far from efficient. To resolve this issue, a constraint is imposed on the design of the ontology. All classes, and so the instances that belong to such classes, must incorporate a datatype property called “name” containing the unique identifier of the instances, simulating the primary key of a database. In this way, if two instances of the same class have the same identifier the system can conclude that both instances are referencing to the same concept.

*(3) Object Properties.* The object properties are set after all the instances have been created. The system distinguishes between two types of relationships: those that occur between instances that belong to classes in the same class group, namely, “same class group relations,” and those relationships established between instances in different groups, namely, “different class group relations.”

In order to establish the relationships between the instances, the system performs the following steps.

1. First, it identifies the closest class group for each row.
2. Then, the system creates the corresponding instances as it was explained before.
3. Third, the system looks for object properties between the classes in the class group and establishes the relationships between the previously created instances.
4. Finally, the system examines the object properties between the classes in different class groups and establishes the corresponding relationships.

### 3.2. Financial Ontology

The need to manage financial data has been coming into increasingly sharp focus for some time. Years ago, these data sat in silos attached to specific applications in banks and financial companies. Then, the Web entered the arena, generating the availability of diverse data sets across applications, departments, and other financial entities. However, throughout these developments, a certain underlying problem has remained unsolved: data reside in thousands of incompatible formats and cannot be systematically managed, integrated, unified, or cleansed. To make matters worse, this incompatibility is not limited to the use of different data technologies or to the multiple different “flavours” of each technology (e.g., the different relational databases in existence), but also its incompatibility in terms of semantics. Thus, the financial domain is becoming a knowledge intensive domain, with a huge number of businesses and companies hinging on it and with a tremendous economic impact on our society. Consequently, there is a need for more accurate and powerful strategies for financial data management. Heedless of the complexity of the domain, financial companies and end-users deem as absolutely necessary a full-fledged integrated approach to cope with the ever-increasing volume of information outperforming current approaches such as Yahoo Finance.

Semantic Technologies are currently achieving a certain degree of maturity. They provide a consistent and reliable basis to face the aforementioned challenges, aiming at a fine-grained approach for organization, manipulation, and visualization of the financial data [52]. In the last few years, several finances-related ontologies have been developed. The ontology TOVE (Toronto Virtual Enterprise) [53], developed by the Enterprise Integration Laboratory from Toronto University, describes a standard organization company as their processes. BORO (Business Object Reference Ontology) is intended to be suitable as a basis for facilitating, among other things, the semantic interoperability of enterprises' operational systems. [54] The consortium DIP (Data Information and Process Integration) developed an ontology for the financial domain which was mainly focused on describing Semantic Web services in the stock market domain [55]. The XBRL Ontology Specification Group developed a set of ontologies for describing financial and economical data in RDF for sharing and interchanging data. This ontology is becoming an open standard means of electronically communicating information among businesses, banks, and regulators [56].

For the purposes of this use case scenario, we have developed a financial ontology based on the ontologies referred to above. The ontology, created from scratch has been defined in OWL. In Table 1, some metrics concerning the financial ontology are presented.

**Figure 7:**
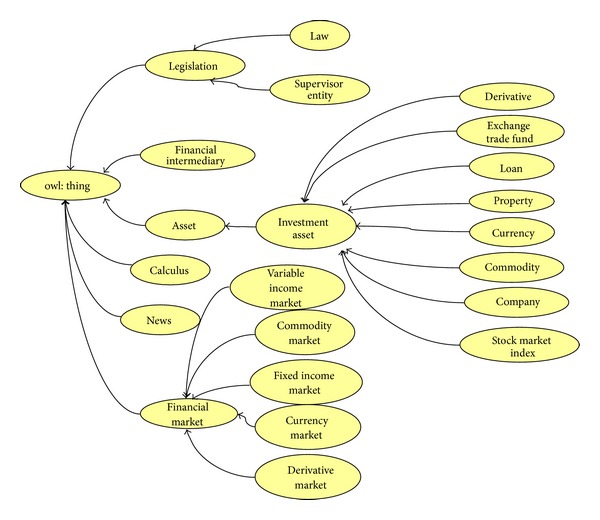
Excerpt of the financial ontology.

The ontology covers four main financial concepts (see Figure 7).

- A financial market is a mechanism that allows people to easily buy and sell financial assets such us stocks, commodities, and currencies. The main stock markets such as Nasdaq, London Stock Exchange, or Madrid Stock Exchange have been modeled in the ontology as subclasses of Stock Market class.
- The concept financial intermediary represents, among other things, the entities that typically invest in the financial markets. Examples of such entities are banks, insurance companies, brokers, and financial advisers.
- The Asset class represents everything of value in which an intermediary can invest, such as stock market indexes, commodities, companies, and currencies. So, for instance, enterprises such as General Electric or Microsoft belong to the company concept and currencies such as the US dollar or Euro are included as individuals of the currency concept.
- The legislation concept comprises the entities that are in charge of supervising the stock market (e.g., the Federal Reserve or the International Monetary Fund) and the regulation and laws that can be applied to the financial domain.

### 3.3. Query System

The query system will show the user all the information stored by the system through a guided query interface.

For the general public to be able to exploit the advantages of the Semantic Web, it is necessary to narrow the gap between the end-user and the mathematical-intensive background of the Semantic Web. The approach taken by most researchers to bridge this gap is the use of natural language interfaces (NLIs) [34–36]. NLIs aim to provide end-users with a means to access knowledge in ontologies hiding the formality of ontologies and query languages. Thus, NLIs help users avoid the burden of learning any logic-based language offering end-users a familiar and intuitive way of query formulation. However, the realization of NLIs involves several issues, one of such problems being linguistic variability and ambiguities. In recent years, Controlled Natural Language (CNL) has received much attention due to its ability to reduce ambiguity of natural language.

SONAR uses OWL-Path [57], a CNL-based NLI that assists users in indicating their queries to the system. By merging the knowledge in both question and domain ontologies, OWL-Path suggests to the user how to complete a query. Once the user has finished formulating the natural language query, OWL-Path transforms it into a SPARQL query and issues it to the ontology repository. In the end, the results of the query are shown back to the user.

**Figure 8:**
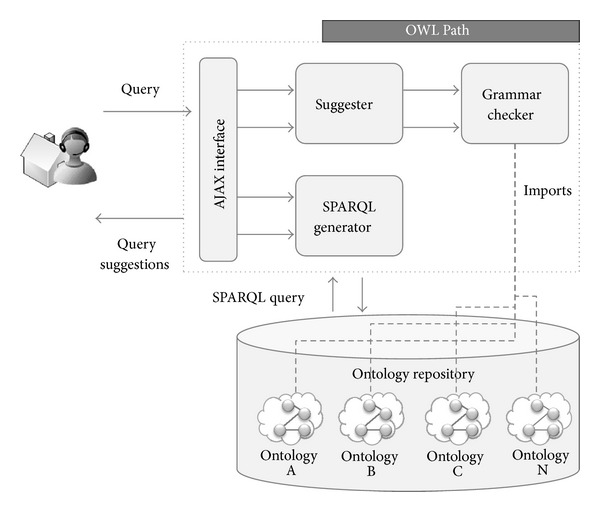
OWL-Path architecture.

The global architecture of OWL-Path is depicted in Figure 8. The system is composed of five main components: the “Ajax interface,” the “Suggester,” the “Grammar checker,” the “SPARQL generator,” and, external to the platform but key to the functioning of the system, the “Ontology repository.” In a nutshell, the system works as follows. Just as the application is started, a set of system ontologies are loaded. Thereafter, users interact with the system through the “Ajax interface”. In order to input a query, users must select the desired terms they want to put next in the sentence from the list of terms provided by the interface. The list of options shown by the “Ajax interface” is generated by the “Suggester” module. In order to generate this list of possible terms, the “Suggester” makes use of the “Grammar checker,” which, by combining the knowledge in both the question and domain ontologies and taking into account the previously inputted terms in the sentence, determines the elements that can come next. At last, when the user completes the query and submits it, the “SPARQL generator” component transforms the natural language sentence into a SPARQL query and issues it to the ontology repository. The results of the query are finally shown back to the user.

Related works (see [10, 34]) use RDF-S ontologies. We use OWL ontologies, which add expressivity to RDF-S. Other research has been conducted that uses OWL for guided input such as GINO [20]. However, they are mostly based on fixed grammars, while the OWL-Path uses a question ontology that permits different ontologies in the ontology repository to be imported and includes restrictions allowed in OWL-DL language.

### 3.4. XBRL Generator

XBRL (Extensible Business Reporting Language) is an open data standard for financial reporting. This format allows information modeling and the expression of semantic meaning commonly used in business reporting. This standard is based on XML and uses XML syntax and related XML technologies such as XML Schema, XLink, Xpath, and namespaces to articulate this semantic meaning.

One of the most important uses of XBRL is to define and exchange financial information, such as a financial statement. The XBRL specification is developed and published by XBRL International, Inc. (XII).

The objective of this paper is to choose one of the most used taxonomies to generate XBRL information about a certain set of companies. In Spain, the National Share Market Commission (NSMC) allows the general public to query or download these taxonomies that contain information about the financial status of a set of companies. However, this group is limited to the companies that belong to the IBEX35 stock market. This limitation can result in the hurdle of having to search the information by oneself if certain financial information must be queried, what is usually presented in the IPP (Spanish acronym of “Public Periodic Information”) taxonomy of XBRL.

For this reason, the objective of this module is to generate XBRL information for IPP taxonomy [58] of those companies that do not belong to a concrete stock market and hence are not generated in an automatic way by the National Share Market Commission. The generation of these information files can also be used, for example, to automatically analyze the generated data in listed firms [59].

The module takes the variables contained in the ontology that should be in the IPP taxonomy such as liquid assets, long- and short-term debts, and financial investments and maps the variables that the ontology manages to the XBRL concepts. Automatically, the system reads the data from the ontology that is stored in OWL format and generates XBRL data following the structure of the taxonomy used (in this case IPP).

**Figure 9:**
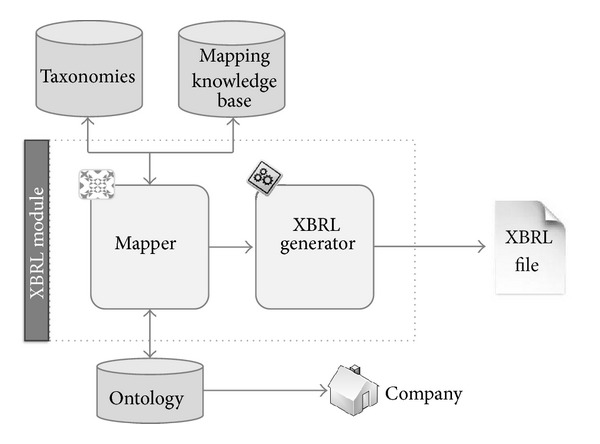
Architecture of XBRL module.

The current system has been developed to support dynamically various types of taxonomies depending on the kind of financial information that you wish to export, but nowadays the system only supports IPP. That means that in the future it will be possible to add other taxonomies and generate configurations to map the existent variables in the ontology of the system to the concepts of the new taxonomies.  Figure 9 shows the internal behavior or architecture of this module.

As it can be observed in this figure, there are two main inputs of this module.

- In the first place is the taxonomy that will be used to generate the XBRL file. As was mentioned before, the current taxonomy that is used and is configured to generate files is only IPP taxonomy, but the system has the capability of managing several taxonomies, thanks to the mapper.
- In second place is the company. The company is necessary in order to access the financial information of that concrete company in the ontology.

The taxonomy is introduced in the system as a code or ID that identifies the ontology in the knowledge base of taxonomies. On the other hand, we also introduce a code or ID to identify the company for which we will generate the financial information to retrieve the data from the ontology.

The mapper is one of the main parts of this module. This piece is able to map the concepts that are stored in the ontology to the concepts that belong to the concrete XBRL taxonomy used. The taxonomy knowledge base in fact contains information about the variables that are stored in the ontology and how they can be mapped to the current taxonomy. The mapper will obtain the data of the company from this knowledge base and from the ontology and send this information to the XBRL generator.

The problem addressed by the mapper solves two basic problems. The first one related to our system is the problem of mapping concepts which comes from a nonstandard ontology and representation structure to XBRL concepts. The second one allows solving the problem of interoperability between heterogeneous systems. This problem can be addressed from several points of view like taxonomy alignment [60, 61]. However, in our case we propose a method based in the use of mapping relations to achieve this problem.

The mapping process is a three-step task which is part of an iterator process that is executed so many times as elements are needed to map. If we have to map, for example, 50 concepts, this process will be executed 50 times and all the steps are obligatory. In this mapping process three elements are used.

- *Ontology* contains the data which the system is going to map to the XBRL financial format.
- *Taxonomies* define the structure of the taxonomy that will be applied to generate the XBRL document based on the information stored in the ontology. The current work is based on IPP taxonomy but the idea is that the mapper should be able to map further taxonomies.
- *Mapping knowledge base* forms part of the mapper module. It is a knowledge base (in our case is based on a database) which contains how the mapping process will be done (through relation definitions). The idea of this knowledge base is the definition of a financial concept which comes from the company financial information (and hence from the ontology provided by the ontology module) and how this concept should be represented in the selected taxonomy from taxonomies module. This is done by establishing a relation between the original concepts (from the ontology) and the mapped concepts (from the XBRL concrete taxonomy).

**Figure 10:**
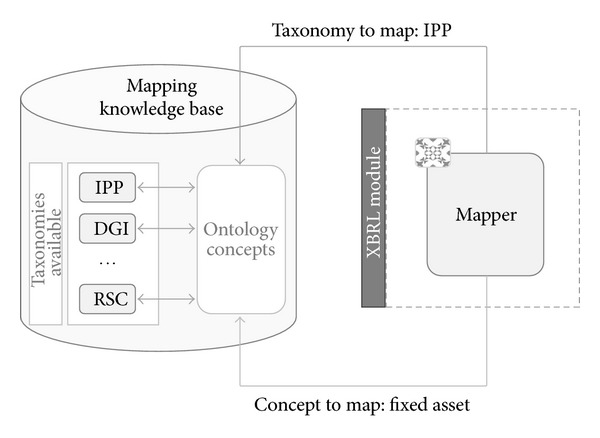
Mapping process.

As it was mentioned before, this process is a three-step task. The steps of this mapping process are the following (Figure 10 depicts this process with an example).

1. The first step consists in the reading of all the elements/concepts which are in the financial ontology of the company that are going to be mapped. This step can be done in two ways depending on how the financial concepts are represented. The type of representation scheme in the ontology can be established in the own ontology. If it is not specified (by a label) the mapper system will try to get the scheme representation through an analysis of the ontology which consists in trying to get the values associated with the concepts through the data properties and if they return a null value assume that the data is stored on an instance. If the analysis returns that other scheme was applied the mapping process will end returning a negative result. The two schemes of representation allowed are the following.
  - *Instance Representation*. If they are represented by instances (an instance represents the value of a concrete concept on a concrete company) the process carried consists in listing the individuals of the ontology. In this scheme each concept of the ontology is represented by a class, and the instance of each class will contain the value associated the concept.
  - *Property Representation*. If the financial concepts are represented as properties (datatype), the process consists in, through the instance which represents the concrete company, reading all the properties associated with concepts and their values.
2. Once the concepts have been loaded in memory by the mapping system (with their respective values), this module will process one by one all the concepts loaded by querying the mapping knowledge base to determine whether the concept can be mapped or not. This information consists in a particular SQL Query where the following two parameters are needed.
  - *Concept*. The first parameter needed is the concept that the system wants to map.
  - *Taxonomy*. The second parameter consists in the name/ID of the taxonomy which will be applied to generate the XBRL file. If the taxonomy changes, the structure of XBRL document can change and the mapping can be different.
3. Once the mapping knowledge base was queried and assuming that a mapping element exists for the concept and the taxonomy provided, the mapper adapts the original data structure of the concept to the XBRL data structure of the associated taxonomy.

One important characteristic of the mapper is that it is not only able to make the mapping based on the “structuration of the information” from the ontology structure to the XBRL taxonomy structure. If, for example, some kind of conversion that should be done exists (imagine that a concrete numerical concept which comes from the ontology needs to be multiplied for a constant in the XBRL format) the mapper will do it. To make this possible, the mapper queries the mapping knowledge base and asks if the concept, for the selected taxonomy, needs some kind of conversion. If the conversion is needed, the mapper will call a concrete transformation/adaptation class/method through dynamic execution in order to convert the concept value to the one specified in the mapping knowledge base.

Finally, the XBRL generator is the software component capable of generating XBRL information. This part can be seen as a simple XML writer, but, in this case, using the specifications of the taxonomy used.

## 4. Evaluation

The subsequent section describes the evaluation of SONAR. This section includes an explanation of the research design throughout. Subsequently, the sample is described along with results of the test. Finally, a discussion of the results is provided.

### 4.1. Research Design

The evaluation of this research proposal was required in order to determine its level of accuracy. The aim of this study is to find out if SONAR provides good results in the construction of XBRL files, taking this information from free access resources available in the Web. Taking this into account, twenty organizations from Spain were selected from the ones that are not included in the stock market and which provide relevant and unstructured information to build XBRL files. All of them were provided to SONAR and, once the system produced XBRL files, this was compared to the output of this process performed in a manual way by four experts (each of them completed 5 files describing 5 companies). These comparisons included two different tests. On the one hand, there is a quantitative test in which Sonar XBRL files and XBRL generated by experts item by item are compared. Each XBRL includes 57 items (and 21 more calculated from these values that are not taken into account). On the other hand, there is a qualitative report for each company in which for every error detected, the expert must explain the nature of the error and its possible sources. This qualitative analysis was carried out with the help of the qualitative data analysis software NVIVO 2.0 (International QSR Pty Ltd.).

### 4.2. Sample

The sample was composed of twenty companies from Spain. None of them are being valued in stock markets and none of them were ever in that particular situation. All of them are IT companies from all over Spain. Seven of them are from Madrid, four from Catalonia, three from Valencia, two from the Basque Country, two from Galicia, and two from Andalucía. In order to guarantee the availability of economic data, all companies in the sample were established before 2006. Data was collected for the 2008 fiscal year in December 2009 and analyzed in January 2010.

With respect to the human sample, four experts were recruited. All of them have a B.S. degree in Economics and were pursuing an MBA. The sample was composed of 2 women and 2 men, with an average age of 27.3.

### 4.3. Results and Discussion

#### 4.3.1. Quantitative Study

The results of the tests, which were carried out on printed copies, were subsequently coded in the statistical analysis tool SPSS. According to the sample, a total of 1,140 items must be detected and coded in XBRL. Results of the process including data from experts and SONAR can be found in Table 2.

As can be derived from results in Table 2, the experts can find 93.77% of the relevant information and SONAR 83.07% of this information. To evaluate the accuracy of SONAR, we used the standard recall, precision, and F1 measures. Recall and precision measures reflect the different aspects of annotation performance. These measures were first used to measure an information retrieval system by Cleverdon et al. [62]. The F1 measure was later introduced by van Rijsbergen [63] in order to combine precision and recall measures, with equal importance, into a single parameter for optimization. The use of these measures is not new in crawlers testing [64–67].

Precision, recall, and F1 measures are defined as follows:

(2) $$\begin{array}{c} \text{Precision}=\frac{\text{Categories}\;\text{found}\;\text{and}\;\text{correct}}{\text{Total}\;\text{Categories}\;\text{Found}},\\ \text{Recall}=\frac{\text{Categories}\;\text{found}\;\text{and}\;\text{correct}}{\text{Total}\;\text{Categories}\;\text{Correct}},\\ \text{F}1=\frac{\left(2*\text{Precision}*\text{Recall}\right)}{\left(\text{Precision}+\text{Recall}\right)}. \end{array}$$

Taking this into account, these measures are as follows for SONAR taking as good data the nominal one:

(3) Precision=0.9284,  Recall=0.8307,F1=0.8769.

On the other hand, if we assume as a standard the data detected by experts, results are as follows:

(4) Precision=0.9284,  Recall=0.8859,F1=0.9067.

A quick look at the results gives the obvious impression that the combined measure is better for the second case. Incidentally, experts did not detect 71 pieces of data from XBRL files and these data pushed down recall and F1 a bit. In both cases the fraction of retrieved XBRL items that are relevant remains unchanged while the fraction of relevant XBRL items that are retrieved changes. However, these measures are more than acceptable compared to other Semantic Technologies crawlers (e.g., [64, 68, 69]).

However, a deeper analysis of results brings improved views.  Table 3 shows results of the expert and SONAR findings in two groups. The first one includes balance sheet items (36) and the second one includes only income statement items (21).

A quick look at results reveals that balance sheet item identification is perfect both for experts and for SONAR. Hence, income statement items identification scores are dramatically different. In this scenario, new precision, recall, and F1 measures taking into account only the income statement items provide these results for SONAR:

(5) Nominal  Data.  Precision=0.7567,Recall=0.5405,  F1=0.6306Experts  Data.  Precision=0.7567,Recall=0.6504,  F1=0.6995.

**Figure 11:**
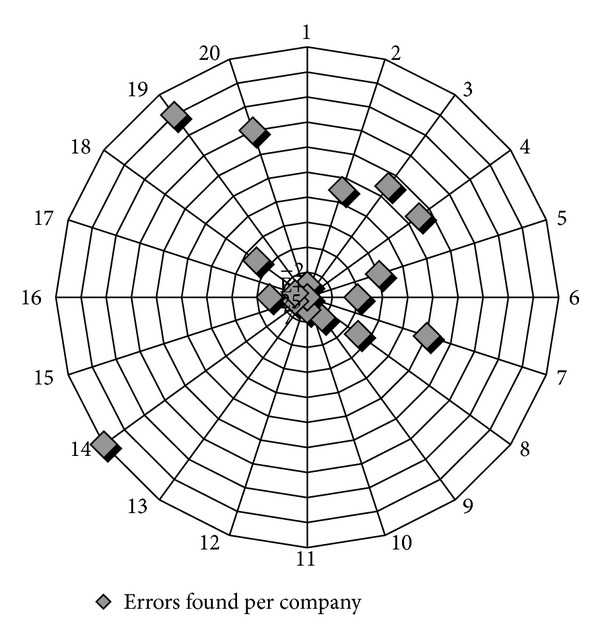
Error frequencies in companies in SONAR.

In order to find out if there are differences among companies in errors detected (122), Figure 11 shows error frequencies for examined enterprises. An error can be defined as a discrepancy between a SONAR generated XBRL item and an expert generated XBRL item.

Taking into account data provided in Figure 11, we can clearly conclude that 76.22% of the total errors found is concentrated in several companies (2, 3, 4, 7, 14, 19, and 20). A first look at qualitative results reveals a concentration of errors in some enterprises and in income statement items, but in order to find out the sources of errors an explanation of the qualitative results must be provided.

#### 4.3.2. Qualitative Study

The objective of this qualitative study is to find out the main reasons for errors detected in the evaluation of SONAR. To do so, after the questionnaires were filled out by experts they were coded in NVivo which helps to look for coherent categories of errors. A total of 122 error reports were included from sample.  Table 4 summarizes the participants descriptions of the source of errors.

A total of 56.6% of the errors provides no data and the rest provides incorrect data. According to the experts' descriptions, only 24.5% of the errors are unknown.

The most important category according to its presence is “No data. Item description mismatching.” A description of the experts shed light on this particular problem: “Data provided in this website has their own particular format and nomenclature that is not easy to match with XBRL items.” A possible solution to this mismatching for SONAR is to provide a broader and more open description of the concepts in order to let the crawler locate and use these items in a proper way.

The second category in importance is “Incorrect data. Data corresponding to other item.” According to experts, many companies publish their financial information using “bizarre” formats that can be decoded “only after a very time consuming task.” Thus, a possible solution to this can be to expand the capacities of the parser to include improved TSiR features. Lastly, the category “Incorrect data. Data corresponding to other year” presents the same problems as the previous one, which can be partially improved using same the methods.

Taking into account the results, the performance of SONAR is more than satisfactory. It is a fact that perfect XBRL construction must be reached, but creating balance sheet items in the correct way is really a significant result. The lack of precision within the income statement can be, in a sense, a result of an incoherent publication format of the companies. There is also a way of improvement for SONAR via a better description of XBRL items and improved TSiR features.

## 5. Conclusions and Future Work

Since the advent of the global economic crisis, the need for accurate, reliable but also ever-growing financial knowledge has become vital for Financial Information Systems with a critical impact in markets. In addition, the use of widespread standards of representation of financial information, such as XBRL language, has gained momentum and forced traditional analysis, design, and development of such systems towards its use.

In this work, we have fundamentally extended and complemented the previous work envisaged with regards to the Semantic Financial Search Engine (SONAR), a conceptual umbrella for a set of efforts and projects funded by both the EU and the Spanish Government, which has proven to be a beneficial Intelligent Financial Information System based on cutting-edge technologies such as Semantic Technologies, natural language processing (NLP), and knowledge representation. The results forthcoming from this followup are threefold. First, we have relied extensively on data crawlers in order to capture useful information from data silos spread all over the Web. Secondly, the role of NLP as an Ontology Population basis together with the benefit of logics as an underlying formal system of the software platform has been validated through the improvements at the implementation and evaluation viewpoint. Finally, the use of the XBRL language has implied a tremendous effort in terms of the standardization and interoperability of the SONAR extension regarding potential integration with other highly related Financial Information Systems.

To sum up, our approach has been deemed a significant step forward toward progress in Intelligent Financial Information Systems, which is being validated by a number of industrial alliances and real-world scenario validation and which will be complemented by an ambitious future work plan. This setup includes the testing of different formalisms which could yield more expressivity than the ones underlying our current approach and also the use of Software-as-a-Service (SaaS) and cloud computing-based strategies to increase the amount of data extracted, managed, and stored, peering into large data management systems and data intensive techniques.

## Figures and Tables

**Algorithm 1:**
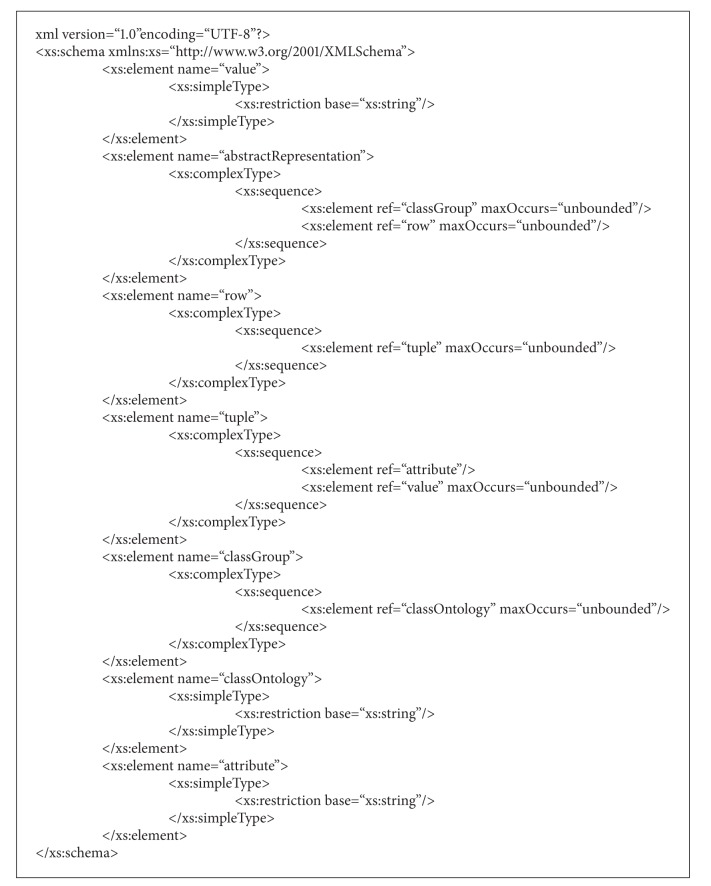
XML Schema of the internal representation format.

**Algorithm 2:**
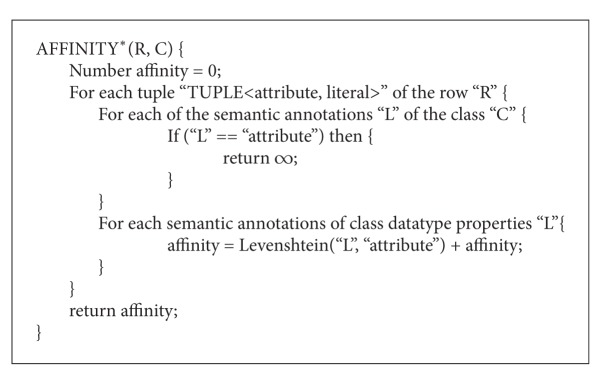
Affinity algorithm.

**Algorithm 3:**
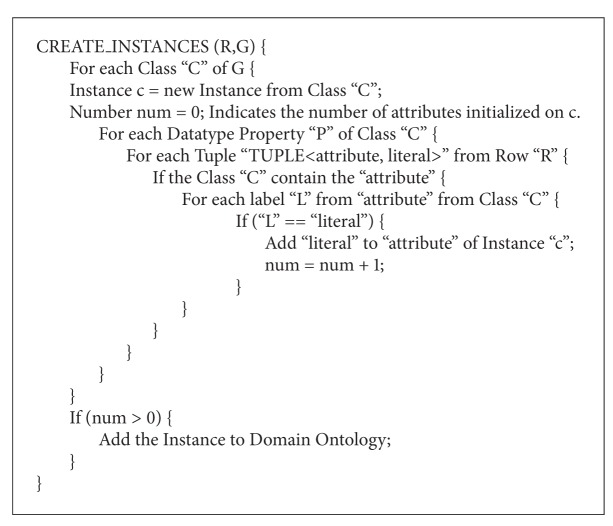
Algorithm to create instances of a particular class or group of classes.

**Table 1:** Details of the financial ontology.

Classes	123
Subclass of properties	86
Datatype properties	72
Object properties	16
Restrictions	87

**Table 2:** Nominal, expert, and SONAR items identification results.

	Nominal	Expert	SONAR	
			Found	Correct
Results	1140	1069	1020	947

**Table 3:** Nominal, expert, and SONAR items identification results divided into balance sheet and income statement items.

	Nominal	Expert	SONAR	
			Found	Correct
Balance sheet	720	720	720	720
Income statement	420	349	300	227
Joint	1140	1069	1020	947

**Table 4:** Descriptions of errors given by participants.

Source	No. of errors found
No data. Item description mismatching	48
No data. Unknown source	21
Incorrect data. Data corresponding to other item	28
Incorrect data. Data corresponding to other year	16
Incorrect data. Unknown source	9
Total	**122**
